# LATER-LIFE EXPERIENCES OF VETERANS AND THEIR CAREGIVERS

**DOI:** 10.1093/geroni/igad104.0160

**Published:** 2023-12-21

**Authors:** Janet Wilmoth

## Abstract

Military service is a defining life-course event that is consequential for the well-being of older adults and their caregivers. Although there has been increased attention to veteran status differences in later-life experiences, critical gaps in knowledge persist. This symposium features four presentations that address existing gaps from various perspectives. Kurth and colleagues use data from an online survey of community-dwelling combat veterans. They find that Vietnam Veterans exhibit more PTSD symptoms than veterans in two younger cohorts, and observed cohort differences are explained by military and post-service experiences. Spiro and colleagues examine later-adult trauma re-engagement (LATR) across multiple samples of Vietnam-era veterans and find that there is variation in LATR by gender and warzone experience. London and colleagues analyze data from adults aged 50+ in the 2021 National Health Interview Survey and find that: non-combat and combat veterans have more difficulty remembering/concentrating than nonveterans; hearing difficulty mediates some of the association between military service experiences and remembering/concentrating; and combat experiences moderate the relationship between hearing difficulty and difficulty remembering/concentrating. Haverhals and colleagues assess the Redefining Elder Care in America Program (RECAP) piloted recently in two VA health care centers and find that combat veterans and their caregivers reported shortcomings and challenges associated with program participation even though they were satisfied overall. Collectively, these papers underscore the enduring influence of combat exposure on older adults and the importance of innovative health care service models that are designed to provide support to caregivers. This is an Aging Veterans: Effects of Military Service across the Life Course Interest Group Sponsored Symposium.

